# Primary vaginal cancer after hysterectomy for benign conditions: a systematic review of the literature

**DOI:** 10.3389/fonc.2024.1334778

**Published:** 2024-01-29

**Authors:** Jing Qian, Kaoma Gracious, Li Chen, Song Xu

**Affiliations:** ^1^ Department of Gynecology, Affiliated Hangzhou First People’s Hospital, Westlake University School of Medicine, Hangzhou, Zhejiang, China; ^2^ International Education College, Zhejiang Chinese Medical University, Hangzhou, Zhejiang, China

**Keywords:** vaginal cancer, vaginal carcinoma, hysterectomy, systematic review, endometrioid adenocarcinoma

## Abstract

**Background:**

Primary vaginal cancer is a rare condition. Some studies have revealed an increased risk of vaginal cancer among patients who have undergone hysterectomy for premalignant and malignant cervical disease. However, there is limited literature available on primary vaginal cancer following hysterectomy for benign conditions.

**Objectives:**

This review aimed to investigate available evidence on clinical characteristics, treatments, and outcomes of primary vaginal cancer following hysterectomy for benign diseases. Additionally, we provide a case of a patient who developed primary vaginal cancer 10 years after undergoing hysterectomy for abnormal uterine bleeding.

**Search strategy:**

We conducted a comprehensive literature search on PubMed, Scopus, Web of Science using a combination of title and abstract represented by “hysterectomy”, and “vaginal cancer”; “vaginal neoplasm”; and “cancer of vagina”. No article type restrictions were applied.

**Main results:**

Eight studies with a total of 56 cases were included in this review. The main symptom observed was vaginal bleeding. Squamous cancer was found to be the most common type, followed by adenocarcinoma. The majority of vaginal cancer cases occurred approximately 10 years after undergoing hysterectomy. The most common location of the tumor was in the vaginal apex. The management approaches varied and details were available in 25 cases. Among these, 7 cases were treated with radiotherapy alone, 1 case received concurrent chemoradiation therapy, and the of rest of the cases underwent surgery as the primary treatment, with or without additional adjuvant therapy. Data of follow-up was available for 15 cases, with 2 cases resulting in death and 2 cases experiencing recurrence. The other cases were alive and well at the time of considered follow up.

**Conclusion:**

Primary vaginal cancer after hysterectomy for benign conditions is an extremely rare condition. It is essential to have high-level evidence to guide the screening and treatment strategy for this rare condition. A part of women who have undergone hysterectomy for benign disorders can benefit from vaginal cytology evaluation. It is reasonable to postpone the initial screening after surgery and to extend the interval between subsequent screenings. Further retrospective case-control trials are expected to determine which specific subgroups of patients mentioned above might most potentially benefit from screening. The treatment decision for vaginal cancer after hysterectomy is more favorable to radiotherapy-based management rather than surgery. Vaginal endometrioid adenocarcinoma may arise from the malignant transformation of endometriosis. More studies are expected to investigate the correlation between these two diseases.

## Introduction

Primary vaginal cancer is a rare disease that affects the lower genital tract, representing 1-2% of all gynecological malignancies and 10% of all vaginal malignant neoplasms (1). In fact, vaginal cancer is more commonly secondary to malignancies from adjacent sites such as cervix, vulvar or even distant sites such as colon, breast, and pancreas (2). Primary vaginal cancer is a type of cancer that specifically occurs in the vagina, without any evidence of cervical or vulvar cancer, or a prior history of these cancers within the last five years (3). The main cause for vaginal cancer is oncogenic HPV (4), along with a few non-HPV related factors. For instance, antenatal exposure to diethylstilbestrol is associated with primary vaginal clear cell adenocarcinoma (5). The most common type of primary vaginal malignancies is squamous cell carcinoma, which is usually HPV induced, accounting for 90%. Adenocarcinoma and other rare entities like melanoma, sarcoma, and lymphoma (6, 7) are also encountered. The risk of primary vaginal cancer increases with age. More than half of the patients are over 70 years old (8).

Primary vaginal cancer can occur in patients who have had a prior hysterectomy. Researches have shown that the most common reason for a prior hysterectomy is cervical cancer or cervical intraepithelial neoplasia (9, 10), which may be explained by the consistent risk factors among primary vaginal cancer, premalignant cervical lesions, and carcinoma of the cervix. Also, scattered reports have revealed that, primary vaginal cancer occurs in patients who have undergone hysterectomy for benign diseases, which is particularly a rare condition. Due to its rarity, the management of this disease is quiet challenging. Recently, our institution admitted a case of primary vaginal adenocarcinoma which occurred 10 years after hysterectomy for benign uterine disease. This rare case inspired us to explore this specific topic further.

Our study systematically reviewed the global literature on the occurrence of primary vaginal cancer after hysterectomy for benign gynecological diseases. Only a few case reports and retrospective studies with small sample sizes provided detailed clinical information, and there is no consensus on the optimal treatment approach. As a result, we conduct a systematic review to investigate the existing evidence on clinical characteristics, management options and prognosis of primary vaginal cancer in hysterectomized patients for benign conditions. Additionally, we emphasize the need for further research to guide the screening and treatment strategy for this rare condition.

## Case presentation

**Figure 1:**
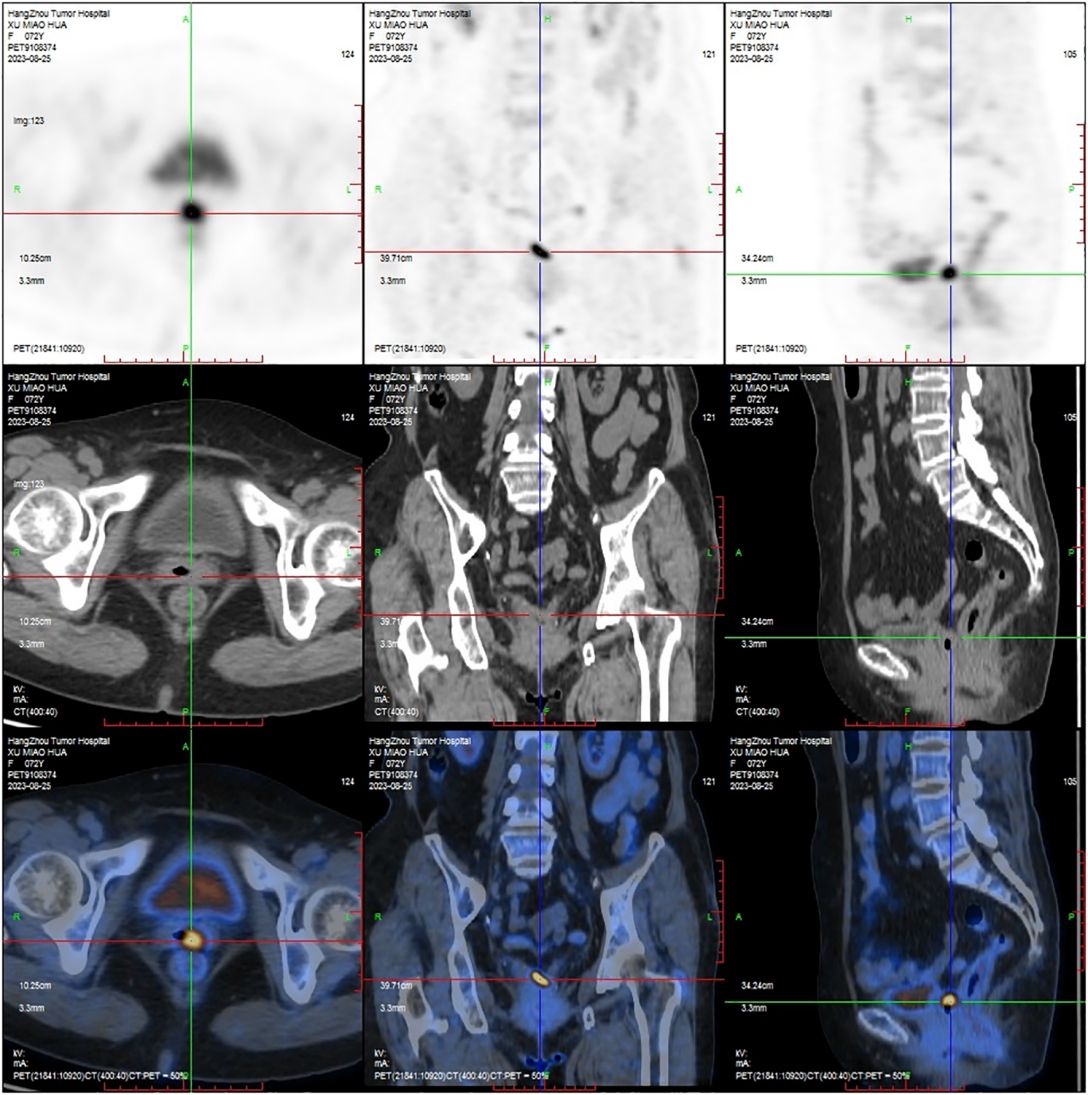
PET-CT depicted the tumor was confined to the apex of vagina.

**Figure 2:**
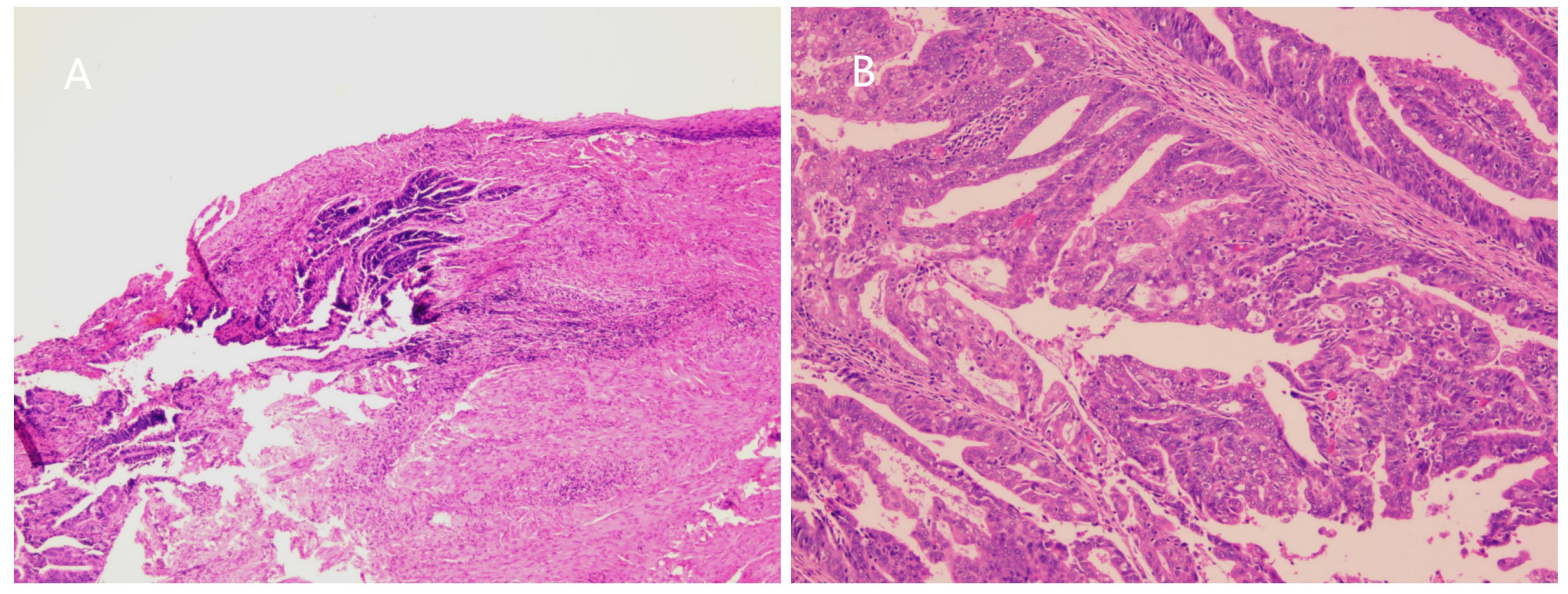
**(A)** Microscopic image showed tumor cells invading vaginal wall (H and E, ×100). **(B)** Microscopic image showed highly differentiated endometrioid adenocarcinoma. (H and E, ×200).

A 72-year-old female, who had a history of hysterectomy and bilateral salpingo-oophorectomy 10 years ago for abnormal uterine bleeding, presented with persistent vaginal spotting for one month in the gynecology department of a local hospital. Upon gynecological examination, a solid ulcerating mass measuring 2*2cm was found at the apex of the vaginal stump. The vaginal stump cytology showed high-grade squamous intraepithelial neoplasia, but human papillomavirus was not detected. Biopsy result indicated endometrioid adenocarcinoma of the vaginal stump. Subsequently, the patient was referred to our hospital for further treatment. PET-CT was scheduled to detect any potential metastatic lesions and determine the initial staging. The result showed that the mass was localized to the vagina (**Figure 1**), without any invasion beyond the vagina or distant spread. The patient was clinically diagnosed with stage I according to the International Federation of Gynecology and Obstetrics staging system (11). Given to the early- stage, small volume, and upper location of the tumor, our medical team planned to perform a radical vaginectomy and pelvic lymphadenectomy under laparotomy, led by an experienced gynecological oncology expert. However, even with such a talented oncologist, the surgery was still challenging. Without the uterus serving as a reliable anatomical marker, it is proved to be difficult to separate the tightly attached vaginal wall from the anterior bladder and posterior rectum. Moreover, the blood supply surrounding the vagina was abundant, and the surgical field of vision was poor, making it hard to stop bleeding. As a result, the patient experienced significant blood loss during the surgery and required a blood transfusion. Eventually, the mass was completely removed along with the vagina and pelvic lymph-nodes. The microscopic examination of the surgical specimen confirmed a highly differentiated endometrioid adenocarcinoma measuring approximately 2.5*1*0.4 cm and infiltrating about half of the vaginal wall (**Figures 2A, B**), without pelvic lymph-nodes involvement. No additional treatment was scheduled after the surgery. The patient remained free of disease during the 3-month follow -up and was recommended to undergo surveillance regularly for evaluation.

## Materials and methods

We performed a systematic search of literature indexed on PubMed, Scopus, and Web of Science (from their inception to September 30, 2023). Our search involved using specific terms in the title and abstract, such as “hysterectomy”, “vaginal cancer”, “vaginal neoplasm”, and “cancer of vagina”. A complete search strategy is provided in **Appendix S1**. Two reviewers (JQ and KG) independently evaluated the titles and abstracts of the records that were retrieved through the database search. The type of articles was not restricted. We only considered articles written in English. We also performed a manual search to include additional relevant articles, by referring to the lists of references in key articles. Full texts of records recommended by at least one reviewer were independently screened by the same two reviewers and assessed for inclusion in the systematic review. Any disagreements between the reviewers were resolved through consensus. Data selection and extraction were carried out according to study type, prior hysterectomy history, histology, intervention, and outcome, using a specifical designed form for capturing information on study characteristics. Data were extracted independently by two authors (JQ and KG) to ensure accuracy and consistency.

## Statistical analysis

For the analysis of outcomes, we calculated the proportions of stage I patients and proportions of vaginal bleeding as the main symptoms amongst all cases. We computed the logarithm of the ratio and its corresponding standard error for each of the studies. A single proportion meta-analysis with inverse-variance weighting was performed using a fixed effects model. Forest plots were created for each outcome, displaying individual study proportions with confidence intervals (CIs) as well as the overall estimate. Heterogeneity was statistically evaluated using the I^2^ test. Statistical analysis was conducted using R packages (v4.1.3).

## Results

### Study assessment

**Figure 3:**
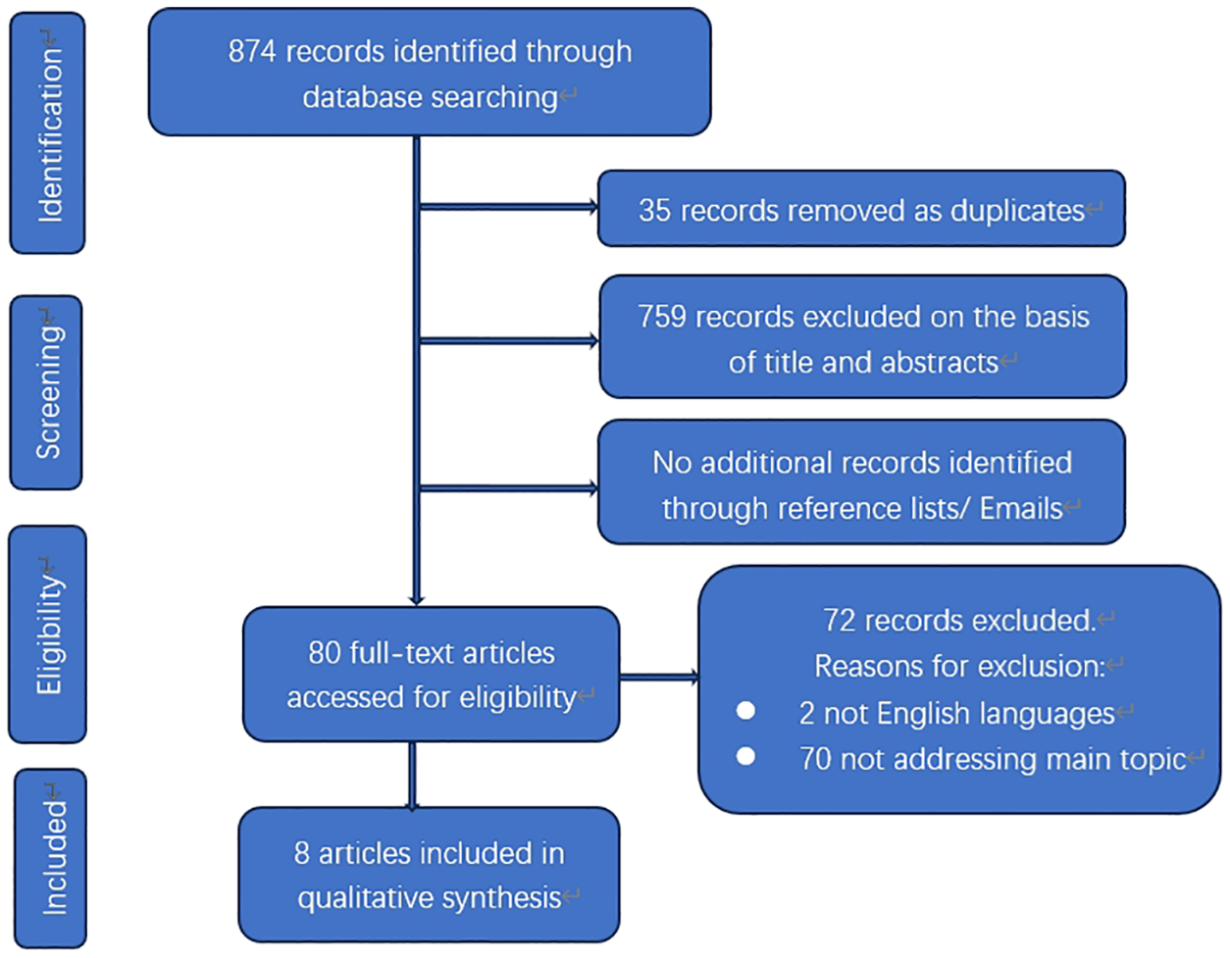
Flowchart of literature selection process.

The electronic database search yielded a total of 874 results (**Figure 3**). After removing duplicates, there were 839 citations left. Among them, 759 were deemed irrelevant to the review based on title and abstract screening. Eighty studies were considered for full-text assessment, and seventy-two were excluded for the following reasons. Two papers were excluded due to being in languages other than English. Seventy papers did not address the main topic or lacked detailed clinical information. In total, 8 studies met the inclusion criteria and were incorporated into the review process (**Table 1**). These papers consist of 5 case reports and 3 small sample sized retrospective analyses published from 1953 to 2022 (12–19).

**Table 1:** Review of primary vaginal cancer after hysterectomy for benign conditions in the literature.

First author	Year	Country	Study design	No. of cases	Age	History of hysterectomy	Symptoms	FIGO stage	Histology	Location	Treatment	Follow-up, months
Nomoto (12)	2010	Japan	Case report	1	57	TH + RSO 15 y before (endometriosis, uterine fibroids)	vaginal discharge	I	Endometrioid adenocarcinoma	Apex	Total abdominal vaginectomy+ PLND + LSO	12months: NED
Kumar (13)	2022	India	Case report	1	40	TH 5 y before (uterine fibroids)	vaginal bleeding	N/A	Mesonephric carcinoma	Apex and anterior	Local resection + brachytherapy	N/A
Stuart (14)	1981	Canada	RA	5	33 to 86	TH an average of 13.1 y before (2 for uterine fibroids, 2 for uterine prolapse, 1 for pelvic inflammatory disease)	Asymptomatic, vaginal bleeding, dysuria	N/A	Squamous cancer	upper third of the vagina (the most common site)	RT	N/A
Dunster (15)	1953	England	Case report	3	49 42 40	TH 6 y before (uterine fibroids) TH 8 y before (uterine fibroids) TH 12 y before (Pelvic inflammatory disease)	Pelvic pain, vaginal bleeding Low backache, vaginal bleeding Pelvic pain, vaginal bleeding	III IV I	Grade I epithelioma Well-differentiated epithelioma well-differentiated epithelioma	Apex Apex Apex	Radium+ X-ray therapy Bilateral excision of the parametrium + BSO + total vaginectomy+ cystectomy Radium	Bony metastases and pelvic recurrence 3 years later N/A 36months: NED
Kusunoki (16)	2018	Japan	Case report	1	54	TH 14 y before (uterine fibroids)	vaginal bleeding	III	Small-cell carcinoma	Apex	Concurrent chemoradiation therapy	12months: NED
Bell (17)	1984	USA	RA	31	37 to 80	27 for TAH 4 for TVH all for benign disease, no detailed description Vaginal cancer occurred: <6 y in 3 patients 6-10 y in 8 patients >10 y in 19 patients Unknown in 1 patient	vaginal bleeding (most common), discharge, pain, asymptomatic	I (11) II (9) III (7) IV (1) Unknown (3)	Squamous cancer (23) Adenocarcinoma (7) Rhabdomyosarcoma (1)	Apex (8) Anterior (4) Posterior (2) Lateral (5) Unknown (12) Upper half (18) Lower half (4) Unknown (9)	N/A	N/A
Staats (18)	2007	USA	RA	13	49 to 81	All patients accepted TAH (4 for uterine fibroids, 4 for endometriosis 2 for abnormal uterine bleeding 3 for other benign diseases) Vaginal cancer occurred: <10 y in 2 patients >10 y in 8 patients Unknown in 3 patients	vaginal bleeding (most common), discharge, obstipation, asymptomatic	I (7) II (4) III (0) IV (2)	Endometrioid adenocarcinoma	Apex (6) Anterior (1) Posterior (2) Lateral (2) Upper (1) Posterior/ Apex/ Lateral (1)	Local resection (7) + RT(2)/Chemo(1)/RT and chemo (1)/None (3) Radical resection (6) + RT(1)/NAC and chemo(1)/ None (2)/ N/A(2)	NED (8) : followed up from 4 months to 6 years Dead of disease (2): 1 for lung metastasis 11 months later 1 for bowel obstruction 9 years later Local recurrence (1): 19 months later N/A (2)
Wolf (19)	2020	USA	Case report	1	72	TH+BSO 25 y before (endometriosis)	vaginal bleeding, discharge	IVA	Endometrioid adenocarcinoma	Apex	pelvic exenteration	N/A

RA, Retrospective analysis; TH, Total hysterectomy; TAH, Total abdominal hysterectomy; TVH, Total vaginal hysterectomy; RSO, Right salpingo-oophorectomy; LSO, Left salpingo-oophorectomy; BSO, Bilateral salpingo-oophorectomy; N/A, not available; PLND, Pelvic lymph node dissection; RT, Radiotherapy; NAC, neoadjuvant chemotherapy; NED, no evidence of disease.

### Main findings

**Figure 4:**
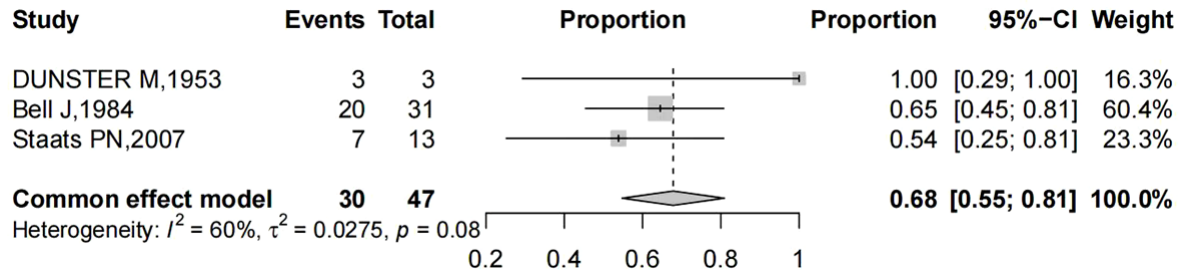
Forest plot showed the proportions of women (with 95% Confidence Intervals) with vaginal bleeding as main symptom among hysterectomized women for benign diseases.

The papers considered included a total of 56 patients. The main characteristics of these studies are listed in **Table 1**. The patients’ age at presentation ranged from 33 to 86 years old. The main symptoms were vaginal bleeding either with vaginal discharge, as well as other uncommon presentations, such as dysuria, obstipation, lower backache, and pelvic pain. Pooling of results from three studies (n = 47 women in whom main symptoms were reported) rendered a summary proportion of 68% (95% CI 26–54) for vaginal bleeding as the main symptom with no significant variation across the studies (I^2^ = 60%, p = 0.08) (**Figure 4**). Vaginal bleeding was the most common chief complaint of the target population and can occur at any stage. However, pelvic pain; backache and obstipation usually presented in late-stage patients. Moreover, some patients may be entirely asymptomatic and were diagnosed during routine examination.

Histopathology showed heterogeneous patterns. By reviewing the study of Bell et al, which included the largest number of patients (31 cases), we found that squamous cell cancer was the most common type of primary vaginal carcinoma in patients who underwent hysterectomy for benign diseases (23 cases) (17), followed by adenocarcinoma (7 cases), and rhabdomyosarcoma (1 case). Another retrospective study with a relatively large sample size by Staats et al. reported 18 cases of primary endometrioid adenocarcinoma of the vagina. Among these cases, 13 had prior hysterectomy for benign diseases, 1 had prior hysterectomy for ovarian endometrioid carcinoma, 2 had prior hysterectomy for unknown reason, and 2 did not have a history of hysterectomy (18). Therefore, 13 cases were included in our review. Stuart et al. reported 29 cases of primary squamous cell carcinoma following hysterectomy. Out of these cases, only 5 had previous hysterectomy for benign reasons and were included in our review (14). The remaining 5 studies in our review were all case reports, which contained 2 cases of endometrioid adenocarcinoma, 1 case of mesonephric carcinoma and 1 case of small-cell carcinoma (12, 13, 16, 19). The report of Dunster et al. was published in 1953, and the pathological type mentioned as “epithelioma” was ambiguous (15).

Among the 56 cases, 16 cases of vaginal cancer occurred within 10 years after the initial hysterectomy. In contrast, 31 cases occurred more than 10 years after. The rest cases did not mention the detailed information.

The most common site of the tumor was the vaginal apex, with 22 out of 56 patients specifically identifying the tumor in that location. The majority of the lesions were located in the upper half of vagina (38 cases). Only 4 cases were presented in the lower half. Valid data was not available for the remaining cases.

**Figure 5:**
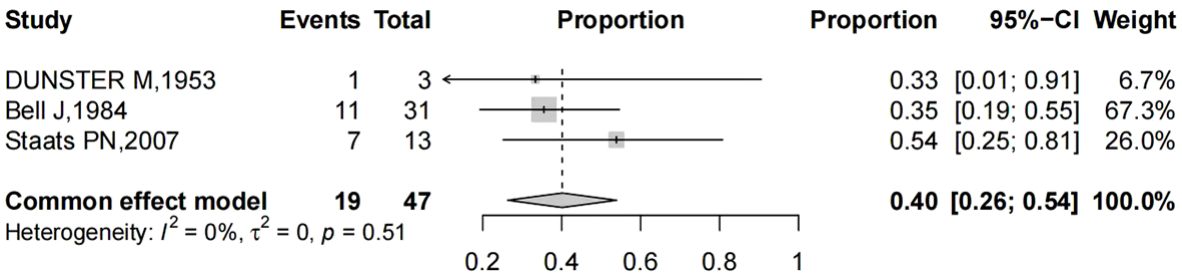
Forest plot showed the proportions of stage I (with 95% Confidence Intervals) primary vaginal cancer among hysterectomized women for benign diseases.

Pooling of results from three studies (n = 47 women with reported stage of vaginal cancer) rendered a summary proportion of 40% (95% CI 26–54) for stage I, showing no significant variation across the studies (I^2^ = 0.0%, p = 0.835) (**Figure 5**).

Treatment strategies were heterogeneous, with relevant details available in 25 cases. Surgery was performed in 17 women (8 case of stage I, 4 case of stage II, 4 case of stage IV, and 1 case without detailed data) followed by additional treatment in 7 cases (4 radiotherapy, 1 chemotherapy, 1 combined radiotherapy and chemotherapy, and 1 combined neoadjuvant chemotherapy and post-operation chemotherapy). Radiotherapy was the primary treatment for eight cases (five squamous cancers, and two were epitheliomas) (14, 15). In one case of small-cell carcinoma, concurrent chemoradiation therapy was performed as the initial treatment (16).

Data on follow-up was available in 15 cases, with follow-up periods ranging from 4 months to 9 years. Overall, 2 deaths were reported (1 due to lung metastasis 11 months later, and 1 due to bowel obstruction 9 years later). Recurrence was reported in 2 patients (1 with bony metastases and pelvic recurrence 3 years later, and 1 with vaginal recurrence 19 months later). All the other women included in the study remained alive and in good health during the follow-up period.

## Discussion

Primary carcinoma of the vagina is a comparatively rare condition, which accounting for only 1-2% of all female reproductive tract cancers (1). In general, the risk factors for primary vaginal carcinomas are the same as those for cervical cancers, with most cases being caused by HPV infection (20). Hysterectomy is one of the most commonly performed surgical procedures in women, with approximately one in nine women undergoing the procedure during their lifetime (21). Although hysterectomy is commonly used as a treatment for gynecologic malignancies, the majority of hysterectomies are actually undertaken for benign gynecologic diseases. Recently, an extremely rare case of primary adenocarcinoma of the vaginal stump was treated in our institution, which emerged 10 years after hysterectomy for benign uterine disease. This case has highlighted our unfamiliarity with the rare disorder and has sparked our interest in getting a deeper understanding of the topic. Therefore, we conducted this systematic review to summarize the relevant literature reports. To the best of our knowledge, this is the first systematic review focusing on the occurrence of primary vaginal cancer after hysterectomy for non-malignant disease. The major strength of our analysis is the robust methodology. However, there are certain limitations that should be acknowledged. Since this topic represents a rare condition, the population of interest is small. Moreover, this systematic review heavily relies on isolated case reports and retrospective analyses with small sample sizes, which may limit the generalizability and robustness of the conclusions.

The role of vaginal vault smears in follow-up of hysterectomized women for reasons other than malignancy has been controversial. The purpose of performing vault smears on asymptomatic hysterectomized women is to detect vaginal intraepithelial neoplasia and prevent vaginal cancer. Opinions regarding the necessity of vault smear have changed over time. There has been a shift from previous enthusiasm to current skepticism, due to that the vaginal intraepithelial neoplasia is 150 times less common than cervical intraepithelial neoplasia, and vaginal cancer is one of the rarest gynecological malignancies (22). A systematic review by Stokes-Lampard et al., which included 6546 hysterectomies for benign diseases elaborated that 1.8% of patients had an abnormal smear, while only 0.12% had an abnormal biopsy, and no vaginal cancers were identified (23). Another evidence-based report by Aldrin et al. revealed that the rate of vaginal cancer and vaginal intraepithelial neoplasia was very low in women with previous hysterectomy for benign conditions (24). Although vaginal intraepithelial neoplasia rate increased in patients with previous cervical intraepithelial neoplasia, even in these patients, vaginal cancer rate was low to 0.01% (24). In 2009, The American College of Obstetricians and Gynecologists stated that, in women who have had a total hysterectomy for benign indications and have no previous history of high-grade CIN, routine cytology screening should be discontinued (25). In our review, 68% of the patients sought for medical help due to vaginal bleeding and were confirmed by biopsy. There’s still a small group of patients who were asymptomatic and presented with abnormalities through vaginal smears. Interestingly, only 40% of patients were diagnosed at stage I. Most cases occurred more than 10 years after hysterectomy. Vaginal vault smear plays a crucial role in the early detection of vaginal cancer. In our opinion, women who have undergone hysterectomy for benign disorders should still receive vaginal cytology evaluation since vaginal cancer can be asymptomatic in its early stages. However, the initial time for screening can be appropriately prolonged after hysterectomy and the intervals between screenings can be lengthened. It might be worthwhile to investigate retrospective case-control trials to determine which specific subgroups of these patients would most potentially benefit from screening.

We observed an attractive finding throughout our review. Among the 56 cases included, 15 cases were histologically diagnosed with endometrioid adenocarcinoma, and the most common reason for a prior hysterectomy among them was endometriosis. We are curious about the potential connection between vaginal endometrioid adenocarcinoma and endometriosis. Previous studies have indicated that women with endometriosis have an increased risk of developing endometrial cancer (26–28). The criteria for defining a cancer arising from endometriosis are as follows: the presence of benign endometrial tissue and cancer in the same site, histology of the tumor consistent with an endometrial origin, and exclusion of metastasis from another primary site (29). Based on these criteria, the vaginal lesion can be diagnosed as vaginal endometrioid carcinoma associated with endometriosis. Staats et al. conducted a study involving 18 cases of primary endometrioid adenocarcinoma of the vagina and identified endometriosis in the tissue adjacent to the carcinoma in 13 cases (18). A review on the malignant transformation of vaginal endometriosis revealed that endometrioid adenocarcinoma (17 out of 37) was the most frequent malignancies arising from endometriosis (30). Therefore, we hypothesize that the residual extrauterine lesion in the vagina after hysterectomy for endometriosis may undergo malignant transformation, most likely leading to endometrioid adenocarcinoma, even though this condition is extremely rare. Further studies are expected to explore the correlation between endometriosis and primary vaginal endometrioid adenocarcinoma.

Given the rarity of vaginal cancer, there are no randomized control trials to guide treatment decisions. The treatment is individualized and depends primarily on histology, tumor volume, anatomical localization of the lesion, stage of the disease, and age of the patient. Different managements can be considered, including surgery, radiotherapy, chemotherapy, or a combination of these approaches. However, the role of surgery in the treatment of vaginal cancer is limited due to the proximity of the vagina to vital organs such as the bladder, urethra, and rectum (31). Therefore, surgery is considered in selected cases as follows: small early-stage tumors that are confined to the upper posterior vagina, late-stage disease with recto-vaginal or vesico-vaginal fistulas, and central recurrence after radiotherapy (1, 7, 32). The type of surgery varies and includes options such as local excision, partial vaginectomy, radical hysterectomy, and pelvic exenteration, usually combined with lymph node assessment. Zhou et al. compared the effectiveness of local excision and vaginectomy for early-stage vaginal carcinoma. They found that vaginectomy resulted in significantly prolonged survival compared to local excision (33). According to FIGO guidelines, for stage I patients with previous hysterectomy involving the upper posterior vagina, a radical upper vaginectomy and pelvic lymphadenectomy are more appropriate (1). Yang et al. elucidated that patients with stage I and II disease had similar survival rates whether treated with surgery or radiation. However, a significant portion of the population required adjuvant radiation therapy after surgery (34). Radiotherapy using external beam and/or brachytherapy is a standard treatment for vaginal cancer, especially in cases that are locally advanced (1, 31, 34, 35). The principal advantage of radiation is organ preservation. A systematic review conducted by Guerri et al. reported that factors associated with better outcomes in the radiotherapy group included early stage of disease, small tumor size (<4 cm), previous hysterectomy, high pre-treatment hemoglobin levels and younger age (35). Another two retrospective studies demonstrated excellent outcomes with definitive radiotherapy, either with external-beam radiation therapy alone or in combination with brachytherapy. These studies also emphasized the importance of individualizing radiotherapy based on patient’s specific factors (36, 37). Nowadays, with the advancements in radiation therapy, image-guided radiotherapy is being used more frequently for the treatment of vaginal cancer, leading to a significant reduction in dose to normal tissue and a decrease in toxicities (38). Chemotherapy is seldom adopted alone in the treatment of vaginal cancer, but rather in combination with other management options. Chemoradiation therapy has shown a rising trend in the treatment of vaginal cancer. A large retrospective cohort study involving 8222 patients demonstrated that chemoradiation was associated with a significant improvement in median overall survival compared to radiation alone (39). Another single institution study including 71 cases highlighted concurrent chemotherapy as a significant predictor of disease-free survival (40). The treatment decisions for vaginal cancer in patients with an intact uterus are not well-established, let alone for those without a uterus. In our review, the managements were heterogeneous without a standard pattern. However, we can draw some insights from the recent case we encountered. The patient, who was confirmed as stage I endometrioid vaginal cancer in the vaginal stump with a small mass, underwent a challenging surgical procedure and experienced significant blood loss. We found that surgical treatment for vaginal cancer after hysterectomy is very difficult, even with an experienced gynecologic oncologist, and can lead to numerous complications. Radiotherapy-based treatment may be a more preferable option for post-hysterectomy vaginal cancer patients.

In conclusion, the occurrence of primary vaginal cancer after hysterectomy of benign diseases is rare, and this is the first systematic review focusing on this topic. Moreover, we present a case of primary vaginal endometrioid adenocarcinoma that occurred 10 years after hysterectomy for a benign condition. Enlightenments from this study are as following: 1. Sometimes, women who have undergone hysterectomy for benign disorders can benefit from vaginal cytology evaluation. But the initial screening time can be properly prolonged after hysterectomy, and the intervals between screenings can be lengthened. Further retrospective case-control trials are expected to determine which specific subgroups of these patients would benefit the most from screening. 2. Vaginal endometrioid adenocarcinoma may arise from malignant transformation of endometriosis. More studies are expected to investigate the correlation between these two diseases. 3. The treatment decision for vaginal cancer after hysterectomy is more favorable to radiotherapy-based management rather than surgery.

## Author contributions

JQ: Conceptualization, Writing – original draft. KG: Data curation, Writing – review & editing. LC: Methodology, Writing – review & editing. SX: Conceptualization, Supervision, Writing – review & editing.
