# Supplementary material for: Primary vaginal cancer after hysterectomy for benign conditions: a systematic review of the literature
Source: Front Oncol. 2024 Jan 29;14:1334778. doi: 10.3389/fonc.2024.1334778 (PMC10859505; doi:10.3389/fonc.2024.1334778)
Supplement: Supplementary file 1 [file Table_1.docx]

| Database | Search Strategy |
| --- | --- |
| PubMed | #1 (((((((((Vaginal Neoplasms[Title/Abstract]) OR (Neoplasm, Vaginal[Title/Abstract])) OR (Neoplasm, Vagina[Title/Abstract])) OR (Cancer, Vaginal[Title/Abstract])) OR (Cancer of the Vagina[Title/Abstract])) OR (Cancer of Vagina[Title/Abstract])) OR (Vagina Cancers[Title/Abstract])) OR (Vagina Cancer[Title/Abstract])) OR (Cancer, Vagina[Title/Abstract])) OR (Cancers, Vagina[Title/Abstract])  #2 hysterectomy [Title/Abstract]  #3 #1 AND #2 |
